# Benefits and Challenges of Current Closed-Loop Technologies in Children and Young People With Type 1 Diabetes

**DOI:** 10.3389/fped.2021.679484

**Published:** 2021-04-30

**Authors:** Julia Fuchs, Roman Hovorka

**Affiliations:** ^1^Wellcome Trust-Medical Research Council Institute of Metabolic Science, University of Cambridge, Cambridge, United Kingdom; ^2^Department of Paediatrics, University of Cambridge, Cambridge, United Kingdom

**Keywords:** diabetes technology, young people, type 1 diabetes (or diabetes), closed-loop insulin delivery, artificial pancreas (AP), children

## Abstract

Recent advances in diabetes technology have led to the development of closed-loop insulin delivery systems for the management of type 1 diabetes. Several such systems are now commercially available for children and young people. While all available systems have been shown to improve glycaemic control and quality of life in this population, qualitative data also highlights the challenges in using closed-loop systems, which vary among different pediatric age-groups. Very young children require systems that are able to cope with low insulin doses and significant glycaemic variability due to their high insulin sensitivity and unpredictable eating and exercise patterns. Adolescents' compliance is often related to size and number of devices, usability of the systems, need for calibrations, and their ability to interact with the system. Given the speed of innovations, understanding the capabilities and key similarities and differences of current systems can be challenging for healthcare professionals, caregivers and young people with type 1 diabetes alike. The aim of this review is to summarize the key evidence on currently available closed-loop systems for children and young people with type 1 diabetes, as well as commenting on user experience, where real-world data are available. We present findings on a system-basis, as well as identifying specific challenges in different pediatric age-groups and commenting on how current systems might address these. Finally, we identify areas for future research with regards to closed-loop technology tailored for pediatric use and how these might inform reimbursement and alleviate disease burden.

Type 1 diabetes is a lifelong, incurable condition characterized by a deficiency of insulin caused by immune-mediated destruction of pancreatic beta-cells in genetically predisposed individuals (1). Its incidence in the pediatric population is increasing by around 3% per year (2), and more than one million children and young people under the age of 20 years are living with the condition worldwide (3). Tight glycaemic control is challenging to achieve, and the majority of children and young people with type 1 diabetes do not meet treatment guidelines for target glycated hemoglobin (HbA1c) (4–7), or only achieve good glycaemic control at the expense of high management burden (8–10). Meeting glycaemic targets is vital, as higher HbA1c levels are associated with an increased risk of premature morbidity and mortality (11–13).

Over the past decades, several new technologies have been developed to improve management of type 1 diabetes, including insulin pumps and real-time continuous glucose monitoring (CGM) devices (14). However, rather than improving, HbA1c levels have worsened in the pediatric age group over the last 10 years (5). While insulin pumps and CGM devices have been shown to lower HbA1c levels and reduce the risk of diabetic ketoacidosis (15–17), they require significant user-input and frequent insulin dosing adjustments to achieve good glycaemic control (10). In recent years, the development of closed-loop systems, which link insulin delivery to sensor glucose levels, have started to transform management of type 1 diabetes (18, 19). These closed-loop systems utilize an algorithm that automatically adjusts insulin delivery via an insulin pump based on real-time sensor glucose levels. This glucose-responsive automated insulin delivery more closely replicates normal physiology. Current hybrid closed-loop systems continue to require user-initiated prandial insulin boluses.

This review summarizes key evidence on current closed-loop systems for children and young people with type 1 diabetes, as well as commenting on user experience and specific challenges in different pediatric age-groups.

## Closed-Loop Systems

Four hybrid closed-loop systems are currently commercially available and licensed for use in children and young people, with varying minimum age for use. These systems are: 670G hybrid closed-loop (HCL) system and 780G advanced HCL (AHCL) (Medtronic, Northridge, California); CamAPS FX interoperable app (CamDiab, Cambridge, UK); and the Control IQ system (Tandem Inc., San Diego, California). Further hybrid closed-loop systems are in development, with pivotal trials under way (20). Table 1 summarizes the key clinical trial evidence of commercialized systems in children and young people.

**Table 1:** Key clinical trials of closed-loop insulin delivery in children and young people using commercialized systems.

**Age group (no. of participants)**	**Duration of closed-loop treatment**	**Type of study**	**Comparator**	**Baseline HbA1c**	**Glycaemic outcomes**	**Year and Key reference**
**Medtronic 670G HCL / 780G AHCL**						
14-21 years (*n* = 30)	3 months (670G)	Single-arm, non-randomized	None	61mmol/mol (7.7%)	TIR 67%, no control arm.	2017 (21, 22)
7-13 years (*n* = 105)	3 months (670G)	Single-arm, non-randomized	None	63mmol/mol (7.9.%)	TIR 65%, no control arm.	2019 (23)
14-29 years (*n* = 113)	3 months (AHCL)	Randomized, crossover	670G HCL	63mmol/mol (7.9%)	TIR 67% with 780G and 63% with 670G (p < 0.0001).	2021 (24)
7-80 years (*n* = 59) [7-13 years (*n* = 19); 14–21 years (*n* = 14)]	4 weeks (AHCL)	Randomized crossover	670G PLGM	60mmol/mol (7.6%)	TIR 70% overall, increased 12 percentage points compared to control (p < 0.001); increased 12 percentage points in 7-13yr olds and 14 percentage points in 14-21yr olds compared to control.	2021 (25)
**Tandem Control IQ**						
6-13 years (*n* = 101)	16 weeks	Randomized, parallel	Sensor-augmented pump	61mmol/mol (7.7%)	TIR 67%, increased 11 percentage points compared to control (p < 0.001).	2020 (26)
14-24 years (*n* = 63)	6 months	Randomized, parallel	Sensor-augmented pump	65mmol/mol (8.1%)	TIR 64%, increased 13 percentage points compared to control (p < 0.001).	2020 (27)
2-5 years (*n* = 12)	3 days	Single-arm, non-randomized	None	56mmol/mol (7.3%)	TIR 71%, no control arm.	2020 (28)
**CamAPS FX (CamDiab)**						
10-18 years (*n* = 12)	3 weeks	Randomized, crossover	Sensor-augmented pump	69mmol/mol (8.5%)	TIR 67%, increased 19 percentage points compared to control (p < 0.001).	2016 (29)
6-65 years (*n* = 86) [6-12 years (*n* = 33); 13-21 years (*n* = 19)]	12 weeks	Randomized, parallel	Sensor-augmented pump	68mmol/mol (8.3%)	TIR 65% overall, increased 11 percentage points compared to control (p < 0.0001); increased 15 percentage points in 6-12yr olds and 14 percentage points in 13-21yr olds compared to baseline.	2018 (30)
1-7 years (*n* = 24)	3 weeks	Randomized, crossover	Closed-loop with diluted insulin U20.	57mmol/mol (7.4%)	TIR 70% closed-loop with U100 and 72% closed-loop with U20, no difference (p=0.16).	2018 (31)

*HCL, hybrid closed-loop; AHCL, advanced hybrid closed-loop; PLGM, predictive low glucose management; TIR, time in range; HbA1c, glycated hemoglobin*.

The closed-loop algorithm is embedded in the software of the tethered insulin pump and communicates wirelessly with the compatible glucose sensor for the Medtronic and Tandem systems (14). For CamAPS FX, the algorithm is embedded in an app, classed as a medical device, residing on an unlocked smartphone that communicates wirelessly with a compatible insulin pump and glucose sensor (14). As more hybrid closed-loop systems become commercially available, it is becoming increasingly complex for people with type 1 diabetes, their families and healthcare professionals to navigate different technologies. While similar in basic principle, there are important differences between each hybrid closed-loop system, and clinicians need to understand key device characteristics in order to provide appropriate clinical support and guidance to children, young people and their families. Several available closed-loop systems now provide online training and education modules, both for healthcare professionals and users, and increasingly module completion is a requirement to allow initiation of closed-loop. Table 2 outlines the capabilities and key similarities and differences of current systems using the CARES paradigm, an educational tool developed by Messer et al. (32), that provides a practical framework to identify key concepts for each closed-loop system. Understanding these key concepts will allow healthcare professionals to set appropriate expectations of hybrid closed-loop system capabilities, and to adjust settings for treatment optimisation to maximize the benefits of this novel therapy.

**Table 2:** Comparison of commercially available closed-loop systems using the CARES paradigm (32).

**CARES**		**670G/780G**	**Control IQ**	**CamAPS FX**
	Licensing	7 years+	6 years+	1 year+
	Availability	USA and Europe	USA & Europe	Europe
	Pump	Medtronic 670G / 780G	Tandem t:slim X2	Dana RS, Dana-i
	Insulin	Rapid-acting	Rapid-acting	Ultra-rapid and rapid-acting
	Closed-loop term	Auto Mode	Control IQ	Auto mode
**Calculate**	Algorithm	Treat-to-target proportional integral derivative with insulin feedback (670G); added fuzzy logic component (780G)	Treat-to-range predictive control	Treat-to-target MPC
	Set-up	TDD, weight, basal rates, ICR, ISF, active insulin time. 7 days of manual mode	TDD, weight, basal rates, ICR, ISF	TDD and weight
	Adaptive learning	Overall	Not specified by manufacturer	Overall, diurnal, meals
	Automated insulin delivery	Based on total daily insulin dose last 2-6 days	Based on pre-programmed basal rates	Based on adaptive learning
	Automated corrections used to supplement basal delivery	670G: No780G: Yes	Yes	No
**Adjust**	Glucose target	670G: Target 6.7mmol/L non-customisable.780G: Customisable target of 5.5, 6.1, or 6.7mmol/L.	Target range 6.2 – 8.9mmol/L.Sleep range 6.2 – 6.7mmol/L.Non-customisable.	Target 5.8mmol/L.Customisable between 4.4-11mmol/L,adjustable in 0.1mmol/L increments.
	Adjustable settings in CL	ICR, active insulin time, glucose target	Basal rates, ICR, ISF, target range	ICR, glucose target
	Non-adjustable in CL	Basal rates, ISF (automatically calculated and adapted)	Active insulin time (set at 5 hours)	Basal rates, active insulin time, ISF (all automatically calculated and adapted)
	Exercise mode	670G: No780G: Yes	Yes	Yes
	Boost mode	No	No	Yes
**Revert**	Sick day rules	—————————————–Recommended to revert to open loop for illness and/or ketones—————————————-		
	Automatically reverts to open loop if…	Prolonged hyperglycemia (670G only), max/min insulin delivery, loss of CGM data, sensor integrity concerns, lack of calibrations.	Loss of CGM data	Loss of CGM data or loss of pump connectivity
**Educate**	Meal bolus	———————————————-Pre-meal bolusing recommended for optimal outcomes———————————————		
		Late bolusing can lead to insulin stacking and hypoglycaemia as CL insulin delivery increases in response to rising glucose		
	Hypo treatment	——————Consider treating hypoglycaemia with fewer carbohydrates depending on recent insulin delivery——————-		
	System optimisation	• System requires finger pricks for HCL functioning • Use of temp target will turn off automated corrections (780G only) • Extended bolus / combo bolus function not available in CL	• Set sleep activity schedule overnight for tighter target • Adjust doses for individuals with shorter active insulin time • Extended bolus possible in CL, max 2 hours	• Use exercise mode “Ease-Off” following hypoglycaemia • Use “Boost” mode during periods of high glucose • Extended bolus / combo bolus function not available in CL
	Online training	No	Yes	Yes
**Sensor**	Type of sensor	Guardian 3	Dexcom G6	Dexcom G6
	Calibrations	670G: 4–6 per day780G: 1–2 per day	Rarely required (factory calibrated)	Rarely required (factory calibrated)
	Sensor life	7 days	10 days	10 days
**Share**	Remote monitoring	No (App in development for 780G)	Yes – Dexcom follow	Yes – Text Alert (Dexcom Follow planned for 2021)
	Upload/Data sharing	Manual downloading	Automated cloud storage for Dexcom data; manual downloading for pump	Automated cloud storage to Diasend (Clarity in 2021)
	Remote bolusing	No	No	Yes

*MPC, model predictive control; CGM, continuous glucose monitor; TDD, total daily insulin dose; ICR, insulin-to-carbohydrate ratio; ISF, insulin-sensitivity factor; CL, closed-loop*.

Clinical trials show that hybrid closed-loop insulin delivery is safe and improves glycaemic outcomes in children of all ages (10, 33), but high quality real-world data remains scarce, particularly for newer systems licensed in the last 12–18 months. Considering the evidence available in terms of system capabilities, qualitative research and observational real-world data, we discuss age-specific challenges in children and young people and how closed-loop systems might address these.

## Toddlers and Young Children

Type 1 diabetes is challenging to manage at any age, but management is further complicated in young children under the age of 7 by a variety of unique physiological, behavioral and developmental factors. Young children have higher variability in insulin requirements and higher insulin sensitivity than older children and adults (34), as well as more unpredictable eating and activity patterns. Furthermore, hypoglycaemia is frequently asymptomatic and can be prolonged, particularly at night-time (35, 36). These factors and resulting parental worry lead to high management burden for parents and caregivers with negative impact on family quality of life (9).

Closed-loop studies in this age-group have been of short duration in small cohorts (Table 1). One of the main benefits of hybrid closed-loop insulin delivery in young children is improved glycaemic control at night-time. In a 3-week closed-loop study in 24 very young children, time in the target range 3.9–10.0 mmol/L was highest overnight with reduction in hypoglycaemia compared to daytime (31) (Table 1). Variability in insulin requirements in young children is highest overnight (34), and closed-loop systems are uniquely positioned to address this by delivering insulin in a glucose-responsive manner. Qualitative data shows that parents noted improvements in quality of sleep with closed-loop, both for themselves and their child (37, 38).

Improvements in glycaemic control are less marked in the daytime, which is likely related to unpredictable eating and activity patterns. Bolus timing is challenging in this age group, as children frequently graze or do not complete meals. Many parents choose to bolus with or shortly after the start of the meal, leading to post-prandial hyperglycaemia (39, 40). The pharmacokinetics and pharmacodynamics of current rapid-acting insulins limit the closed-loop system's ability to mitigate immediate post-prandial hyperglycaemia (41), and the resultant increase in algorithm-driven insulin delivery increases the risk of delayed hypoglycaemia. New ultra-rapid acting insulins, which have faster onset and offset of action, have the potential to address this issue, but these have not been trialed in closed-loop systems in the pediatric age-group.

Another limitation of current closed-loop systems is the minimum total daily insulin dose required for optimal system performance. While this does not preclude use in those young children with a very low total daily dose, it can limit the benefit of closed-loop therapy due to the high variability of absorption with such small volumes (42). A randomized trial comparing closed-loop insulin delivery using diluted and standard strength insulin showed no difference in glycaemic outcomes between the two groups (31). However, only a small number of participants had a total daily insulin dose of <10 units in this cohort. Previous shorter closed-loop studies using diluted insulin in this age group showed reduced inter-individual variability in time to peak insulin action with diluted insulin (42), suggesting that insulin dilution may be beneficial on a case-by-case basis in those with a very low total daily insulin dose.

In spite of these limitations, qualitative studies reported parents spending less time performing diabetes-related activities and feeling less stressed when their child was using closed-loop, resulting in reduced management burden overall (37, 38).

There is limited real-world closed-loop data available for very young children. This is in part due to the fact that closed-loop insulin delivery is only licensed for one commercialized system (CamAPS FX) in this age group, with other systems being used off-license in some centers. A retrospective case series of the 670G HCL system in 16 children under the age of 7 years showed improvements in glycaemic control compared to baseline (43). There was an increase in time in hypoglycaemia, however this was still low at 2.4% (43). Importantly, the results of further clinical closed-loop trials in very young children are expected to be reported in the near future, and should result in licensing of a wider variety of systems in this age group.

## Children

There is significantly more evidence of closed-loop safety and efficacy in school-aged children, compared to those below the age of 7 years. Studies of longer duration in larger cohorts show significant improvements in glycaemic control (26, 30), with no difference in time in hypoglycaemia (Table 1).

Despite their young age, school-aged children often independently manage their diabetes to a significant extent (10). This is in part due to a high turnover of caregiving adults, whose diabetes management knowledge is often minimal (44). This leads parents and children to tolerate higher glucose levels to avoid hypoglycaemia (10, 45), and may limit children's ability to participate in certain activities or events without parental supervision.

Closed-loop systems address this issue in two ways. The automation of insulin delivery in response to real-time sensor glucose levels reduces the need for user input, and events such as post-prandial hyperglycaemia or exercise-induced hypoglycaemia may be prevented or attenuated by the closed-loop system itself. This system-innate responsiveness has the potential to give children more freedom in their activities by increasing parents' confidence in the child's safety. In a qualitative study interviewing parents of children using a closed-loop system, they reported being more willing to allow their child to participate in activities such as school trips or sleepovers than before (21). Secondly, the remote monitoring capabilities of some closed-loop systems give reassurance to parents and children, by allowing parents to adopt a watchful waiting approach, and to intervene and support their child's decision making if required (46). Both parents and children reported closed-loop insulin delivery improving their quality of life and reducing diabetes management burden (21, 38).

While clinical trial evidence shows significant benefits with closed-loop insulin delivery in terms of glycaemic control, parents noted the importance of trusting the closed-loop system for optimum benefit (21). They noted that an initial adjustment period was required, during which they realized that taking action to address low or high glucose levels could be counter-productive to the system's ability to manage glucose levels (21). Additional education around minimizing interventions when using closed-loop insulin delivery could be beneficial when commencing this therapy.

Similar to younger children, real-world closed-loop data is limited. All commercially available closed-loop systems, apart from the 670G HCL system, were only licensed for children in the last 12–18 months. A prospective observational study of people aged 9–61 years using the 670G HCL system for 1 year found that closed-loop use declined significantly over time with a high proportion of closed-loop discontinuation (47). Children and adolescents were more likely to discontinue closed-loop. The main reasons for discontinuation were frequent sensor calibration requirements and a high number of closed-loop exits (47). Another prospective observational study of youth aged 2–25 years using the 670G HCL system showed similar results, with a steady decline of closed-loop use over time (48). Newer generation systems, such as Control IQ and CamAPS FX, using a factory-calibrated sensor alleviate a key reason for closed-loop discontinuation. While the 780G AHCL system still requires sensor calibration, clinical trials show a significant reduction in closed-loop exits (24), suggesting improved usability in this newer iteration.

## Young People

HbA1c levels are highest in young people aged 13–17 years (5). Diabetes self-management is particularly challenging in this age group due to a variety of factors, including peer group influences, importance of body image, less parental oversight, greater risk-taking, and fear of hypoglycaemia, leading to higher levels of diabetes distress (49, 50). Closed-loop insulin delivery offers a novel way to address these issues, although important considerations remain with regards to choice of system for individual users.

Clinical studies have shown that closed-loop insulin delivery significantly improves glycaemic control in this age-group (25, 51), including in those with sub-optimal glycaemic control (30), and that improvements are sustained over time (27, 52). Importantly, qualitative studies of young people using closed-loop and their parents have reported significant improvements in quality of life and reduced diabetes management burden (21, 46, 49, 50).

Fitting in with peers and taking part in normal activities is very important to young people (50), which can lead them to neglect diabetes self-management tasks such as finger prick blood glucose checks and pre-meal bolusing (53). Glucose sensors reduce burden and allow young people to discreetly check glucose levels, as well as allowing glucose-responsive insulin delivery in closed-loop. While sensors requiring calibration can be a significant factor in low closed-loop usage (38, 48, 54), several systems now use factory-calibrated glucose sensors, with high sensor wear reported in clinical trials (52) and reduced device-burden reported in qualitative studies (49). In a qualitative study of young people using closed-loop from onset of diabetes, participants reported that the closed-loop system had helped them continue to lead normal lives despite having diabetes (49) by alleviating the need for disruptive finger pricking and automatically adjusting insulin delivery in response to high or low glucose levels (50, 55).

Current closed-loop insulin delivery systems are all hybrid systems, which require user-initiated prandial boluses for optimal efficacy. However, studies have shown that systems have the ability to cope with missed boluses, while still providing improvements in short-term glycaemic control without an increase in hypoglycaemia (53). Data from a recent 6-month closed-loop study using Control IQ in young people aged 14–24 years showed sustained glycaemic improvements (27), suggesting that closed-loop remains efficacious in a group where there is higher likelihood of sub-optimal compliance (53).

Common barriers to closed-loop insulin delivery in this age group are device burden and alarm frequency (49). Young people preferentially wear devices in non-visible locations (21) and at times avoid activities where devices may be visible to others, such as swimming (49). A system with remote data viewing and bolusing capability via a mobile phone was positively received by young people, as this offered maximum discretion in peer environments (50) while enabling them to make management decisions (21, 49). Audible alarms can negatively impact quality of life, and in a qualitative study of closed-loop in this age group parents reported some young people opting to disconnect from the system when socializing with peers to limit alarms sounding in public (50). Most systems now feature personalisable alarm settings and healthcare providers should support young people in choosing settings that minimize interruptions while providing an adequate safety net.

Real-world data of the first commercially available hybrid closed-loop system, the 670G HCL system, showed high rates of closed-loop discontinuation, due to frequent sensor calibration requirements and a high number of closed-loop exits (47, 48). As described above, newer generation systems have shown much higher closed-loop use and fewer closed-loop exits in clinical trials (24, 27). A recently published real-world study assessed glycaemic control and quality of life by administering questionnaires to more than 1,000 Control-IQ users aged 14 years and over (21). Users reported a positive impact on their quality of life and sleep quality over a 2-month period after starting closed-loop therapy. Minimizing device and alarm burden and enabling easy and discreet user interaction, while maintaining glycaemic benefits should be the main goals of further closed-loop system developments in this age group.

## Future Research

Across all pediatric age groups device burden and connectivity problems remain an issue with regards to closed-loop insulin delivery (49, 50, 55). System-integration with factory-calibrated sensors is paramount to ensure high and sustained closed-loop usage. Connectivity issues resulting in closed-loop exits need to be improved, for example by increasing allowable distance between devices or integrating the algorithm with smart devices, such as watches. Remote data viewing and bolusing capabilities are highly valued by parents of young children, as this minimizes disturbance during sleep or play, and also by young people, where it allows discreet interaction with the closed-loop system, and this should become standard to all commercial closed-loop systems (21). Furthermore, automatic cloud storage and data sharing facilitates interaction with healthcare providers and improves remote consultations, reducing burden (56). Currently, most systems are limited to one pump and CGM model, with specific devices having user-dependent pros and cons. Inter-operable systems, where users can mix and match devices that suit their individual needs, should be the focus of future developments.

The majority of systems are not licensed for very young children, and there is lack of clinical trial evidence with regards to efficacy and safety over longer periods. Clinical guidance is required for those whose total daily insulin dose is below the required threshold for closed-loop operation. Particularly for young people, new faster insulins could provide an increasingly realistic pathway to a more fully closed-loop system, where accurate carbohydrate counting and prandial bolusing is no longer required. Closed-loop studies in the pediatric age-group with ultra-rapid acting insulins are required to assess feasibility and safety.

Due to the novelty of closed-loop insulin delivery technology there is little real-world evidence to guide clinicians and users. Longer-term real-world studies are required to assess whether glycaemic and quality of life benefits are sustained long-term and what features are most desired by users to improve ease-of-use. This will inform system reimbursement, facilitating wider access across the diabetes population. Furthermore, several systems now incorporate personalisable glucose targets, as well as user-initiated modes that reduce or intensify insulin delivery. While users have expressed a wish for more collaboration with closed-loop systems in qualitative studies (49), safety and efficacy of these features needs to be evaluated to help optimize their use.

One of the most important issues facing healthcare providers and the diabetes community is access to closed-loop therapy. Insurance coverage of closed-loop therapy is currently poorly established, and the high cost of CGM and insulin pumps is a significant barrier to uptake for those who cannot afford to self-fund the technology (57). This may lead to growing disparities in those from lower socio-economic backgrounds (57). Future research needs to incorporate robust health economic analysis and should aim to show long-term cost-effectiveness to aid reimbursement for closed-loop therapy.

## Conclusion

Closed-loop insulin delivery improves glycaemic control in all pediatric age groups, while crucially reducing the high management burden associated with this chronic disease, thus improving quality of life for the whole family. Children, young people, and their families now have a variety of commercially available closed-loop systems to choose from, with further systems in development. Future research should focus on improving systems to further reduce diabetes management burden and optimize efficacy, ultimately informing system reimbursement and facilitating wider access across the diabetes population.

## Author Contributions

JF wrote the manuscript. RH edited, critically reviewed, and approved the final submitted version of the manuscript. All authors contributed to the article and approved the submitted version.

## Conflict of Interest

RH reports having received speaker honoraria from Eli Lilly and Novo Nordisk, serving on advisory panel for Eli Lilly and Novo Nordisk; receiving personal fees from BBraun, Medtronic and Abbott Diabetes Care; patents related to closed-loop insulin delivery, shareholder and director at CamDiab Ltd. The remaining author declares that the research was conducted in the absence of any commercial or financial relationships that could be construed as a potential conflict of interest.

## References

[1] Todd JA. Etiology of type 1 diabetes. Immunity. (2010) 32:457–67. 10.1016/j.immuni.2010.04.001 20412756

[2] Patterson CC Harjutsalo V Rosenbauer J Neu A Cinek O Skrivarhaug T. Trends and cyclical variation in the incidence of childhood type 1 diabetes in 26 European centers in the 25 year period 1989-2013: a multicentre prospective registration study. Diabetologia. (2019) 62:408–17. 10.1007/s00125-018-4763-3 30483858

[3] Patterson CC Karuranga S Salpea P Saeedi P Dahlquist G Soltesz G. Worldwide estimates of incidence, prevalence and mortality of type 1 diabetes in children and adolescents: results from the International Diabetes Federation Diabetes Atlas, 9th edition. Diabetes Res Clin Pract. (2019) 157:107842. 10.1016/j.diabres.2019.107842 31518658

[4] Mair C Wulaningsih W Jeyam A McGurnaghan S Blackbourn L Kennon B. Glycaemic control trends in people with type 1 diabetes in Scotland 2004-2016. Diabetologia. (2019) 62:1375–84. 10.1007/s00125-019-4900-7 31104095 PMC6647722

[5] Foster NC Beck RW Miller KM Clements MA Rickels MR DiMeglio LA. State of type 1 diabetes management and outcomes from the T1D Exchange in 2016-2018. Diabetes Technol Ther. (2019) 21:66–72. 10.1089/dia.2018.0384 30657336 PMC7061293

[6] Wood JR Miller KM Maahs DM Beck RW DiMeglio LA Libman IM. Most youth with type 1 diabetes in the T1D Exchange clinic registry do not meet American Diabetes Association or International Society for Pediatric and Adolescent Diabetes clinical guidelines. Diabetes Care. (2013) 36:2035–7. 10.2337/dc12-1959 23340893 PMC3687259

[7] DiMeglio LA Acerini CL Codner E Craig ME Hofer SE Pillay K. ISPAD Clinical Practice Consensus Guidelines 2018: glycemic control targets and glucose monitoring for children, adolescents, and young adults with diabetes. Pediatr Diabetes. (2018) 19(Suppl. 27):105–14. 10.1111/pedi.12737 30058221

[8] Anderzen J Hermann JM Samuelsson U Charalampopoulos D Svensson J Skrivarhaug T. International benchmarking in type 1 diabetes: large difference in childhood HbA1c between eight high-income countries but similar rise during adolescence-A quality registry study. Pediatr Diabetes. (2020) 21:621–7. 10.1111/pedi.13014 32249476

[9] Harrington KR Boyle CT Miller KM Hilliard ME Anderson BJ Van Name M. Management and family burdens endorsed by parents of youth <7 years old with type 1 diabetes. J Diabetes Sci Technol. (2017) 11:980–7. 10.1177/1932296817721938 28770627 PMC5951003

[10] Sherr JL. Closing the loop on managing youth with type 1 diabetes: children are not just small adults. Diabetes Care. (2018) 41:1572–8. 10.2337/dci18-0003 29936422 PMC6054496

[11] Diabetes Control Complications Trial/Epidemiology of Diabetes Interventions Complications Study Research Group. Mortality in type 1 diabetes in the DCCT/EDIC versus the general population. Diabetes Care. (2016) 39:1378–83. 10.2337/dc15-2399 27411699 PMC4955932

[12] Ehrmann D Kulzer B Roos T Haak T Al-Khatib M Hermanns N. Risk factors and prevention strategies for diabetic ketoacidosis in people with established type 1 diabetes. Lancet Diabetes Endocrinol. (2020) 8:436–46. 10.1016/S2213-8587(20)30042-5 32333879

[13] Basina M Maahs DM. Age at type 1 diabetes onset: a new risk factor and call for focused treatment. Lancet. (2018) 392:453–4. 10.1016/S0140-6736(18)31811-7 30129445

[14] Leelarathna L Choudhary P Wilmot EG Lumb A Street T Kar P. Hybrid closed-loop therapy: where are we in 2021? Diabetes Obes Metab. (2020) 23:655–60. 10.1111/dom.14273 33269551

[15] Tauschmann M Hermann JM Freiberg C Papsch M Thon A Heidtmann B. Reduction in diabetic ketoacidosis and severe hypoglycemia in pediatric type 1 diabetes during the first year of continuous glucose monitoring: a multicenter analysis of 3,553 subjects from the DPV Registry. Diabetes Care. (2020) 43:e40–2. 10.2337/dc19-1358 31969340

[16] Misso ML Egberts KJ Page M O'Connor D Shaw J. Continuous subcutaneous insulin infusion (CSII) versus multiple insulin injections for type 1 diabetes mellitus. Cochrane Database Syst Rev. (2010). CD005103. 10.1002/14651858.CD005103.pub2 20091571 PMC12582037

[17] Johnson SR Cooper MN Jones TW Davis EA. Long-term outcome of insulin pump therapy in children with type 1 diabetes assessed in a large population-based case-control study. Diabetologia. (2013) 56:2392–400. 10.1007/s00125-013-3007-9 23963323

[18] Bekiari E Kitsios K Thabit H Tauschmann M Athanasiadou E Karagiannis T. Artificial pancreas treatment for outpatients with type 1 diabetes: systematic review and meta-analysis. BMJ. (2018) 361:k1310. 10.1136/bmj.k1310 29669716 PMC5902803

[19] Weisman A Bai J-W Cardinez M Kramer CK Perkins BA. Effect of artificial pancreas systems on glycaemic control in patients with type 1 diabetes: a systematic review and meta-analysis of outpatient randomized controlled trials. Lancet Diabetes Endocrinol. (2017) 5:501–12. 10.1016/S2213-8587(17)30167-5 28533136

[20] Boughton CK Hovorka R. New closed-loop insulin systems. Diabetologia. (2021) 64:1007–15. 10.1007/s00125-021-05443-1 33550442 PMC8012332

[21] Lawton J Blackburn M Rankin D Allen JM Campbell FM Leelarathna L. Participants' experiences of, and views about, daytime use of a day-and-night hybrid closed-loop system in real life settings: longitudinal qualitative study. Diabetes Technol Ther. (2019) 21:119–27. 10.1089/dia.2018.0306 30720338 PMC6434584

[22] Garg SK Weinzimer SA Tamborlane WV Buckingham BA Bode BW Bailey TS. Glucose outcomes with the in-home use of a hybrid closed-loop insulin delivery system in adolescents and adults with type 1 diabetes. Diabetes Technol. Ther. (2017) 19:155–63. 10.1089/dia.2016.0421 28134564 PMC5359676

[23] Forlenza GP Pinhas-Hamiel O Liljenquist DR Shulman DI Bailey TS Bode BW. Safety evaluation of the MiniMed 670G System in children 7-13 Years of age with type 1 diabetes. Diabetes Technol Ther. (2019) 21:11–9. 10.1089/dia.2018.0264 30585770 PMC6350071

[24] Bergenstal RM Nimri R Beck RW Criego A Laffel L Schatz D. A comparison of two hybrid closed-loop systems in adolescents and young adults with type 1 diabetes (FLAIR): a multicentre, randomized, crossover trial. Lancet. (2021) 397:208–19. 10.1016/S0140-6736(20)32514-9 33453783 PMC9194961

[25] Collyns OJ Meier RA Betts ZL Chan DSH Frampton C Frewen CM. Improved glycemic outcomes with Medtronic MiniMed Advanced Hybrid Closed-Loop delivery: results from a randomized crossover trial comparing automated insulin delivery with predictive low glucose suspend in people with type 1 diabetes. Diabetes Care. (2021) 44:969–75. 10.2337/figshare.13618910 33579715

[26] Breton MD Kanapka LG Beck RW Ekhlaspour L Forlenza GP Cengiz E. A randomized trial of closed-loop control in children with type 1 diabetes. N Engl J Med. (2020) 383:836–45. 10.1056/NEJMoa2004736 32846062 PMC7920146

[27] Isganaitis E Raghinaru D Ambler-Osborn L Pinsker JE Buckingham BA Wadwa RP. Closed-loop insulin therapy improves glycemic control in adolescents and young adults: outcomes from the international Diabetes Closed-Loop (iDCL) trial. Diabetes Technol Ther. (2021). 10.1089/dia.2020.0572. [Epub ahead of print]. 33216667 PMC8080922

[28] Ekhlaspour L Schoelwer MJ Forlenza GP Deboer MD Norlander L Hsu L. Safety and performance of the Tandem t:slim X2 with Control-IQ automated insulin delivery system in toddlers and preschoolers. Diabetes Technol Ther. (2020). 10.1089/dia.2020.0507. [Epub ahead of print]. 33226837 PMC8080923

[29] Tauschmann M Allen JM Wilinska ME Thabit H Acerini CL Dunger DB. Home use of day-and-night hybrid closed-loop insulin delivery in suboptimally controlled adolescents with type 1 diabetes: a 3-week, free-living, randomized crossover trial. Diabetes Care. (2016) 39:2019–25. 10.2337/dc16-1094 27612500 PMC5079605

[30] Tauschmann M Thabit H Bally L Allen JM Hartnell S Wilinska ME. Closed-loop insulin delivery in suboptimally controlled type 1 diabetes: a multicentre, 12-week randomized trial. Lancet. (2018) 392:1321–9. 10.1016/S0140-6736(18)31947-0 30292578 PMC6182127

[31] Tauschmann M Allen JM Nagl K Fritsch M Yong J Metcalfe E. Home use of day-and-night hybrid closed-loop insulin delivery in very young children: a multi-center, 3-week, randomized trial. Diabetes Care. (2019) 42:594–600. 10.2337/dc18-1881 30692242

[32] Messer LH Berget C Forlenza GP. A clinical guide to advanced diabetes devices and closed-loop systems using the CARES paradigm. Diabetes Technol Ther. (2019) 21:462–9. 10.1089/dia.2019.0105 31140878 PMC6653788

[33] Dovc K Battelino T. Closed-loop insulin delivery systems in children and adolescents with type 1 diabetes. Expert Opin Drug Deliv. (2020) 17:157–66. 10.1080/17425247.2020.1713747 32077342

[34] Dovc K Boughton C Tauschmann M Thabit H Bally L Allen JM. Young children have higher variability of insulin requirements: observations during hybrid closed-loop insulin delivery. Diabetes Care. (2019) 42:1344–7. 10.2337/dc18-2625 31221700 PMC6609966

[35] Wiltshire EJ Newton K McTavish L. Unrecognised hypoglycaemia in children and adolescents with type 1 diabetes using the continuous glucose monitoring system: prevalence and contributors. J Paediatr Child Health. (2006) 42:758–63. 10.1111/j.1440-1754.2006.00973.x 17096709

[36] Juvenile Diabetes Research Foundation Continuous Glucose Monitoring Study G. Prolonged nocturnal hypoglycemia is common during 12 months of continuous glucose monitoring in children and adults with type 1 diabetes. Diabetes Care. (2010) 33:1004–8. 10.2337/dc09-2081 20200306 PMC2858162

[37] Musolino G Dovc K Boughton CK Tauschmann M Allen JM Nagl K. Reduced burden of diabetes and improved quality of life: experiences from unrestricted day-and-night hybrid closed-loop use in very young children with type 1 diabetes. Pediatr Diabetes. (2019) 20:794–9. 10.1111/pedi.12872 31140654 PMC6771658

[38] Barnard KD Wysocki T Ully V Mader JK Pieber TR Thabit H. Closing the loop in adults, children and adolescents with suboptimally controlled type 1 diabetes under free living conditions: a psychosocial substudy. J Diabetes Sci Technol. (2017) 11:1080–8. 10.1177/1932296817702656 28367636 PMC5951034

[39] Streisand R Monaghan M. Young children with type 1 diabetes: challenges, research, and future directions. Curr Diab Rep. (2014) 14:520. 10.1007/s11892-014-0520-2 25009119 PMC4113115

[40] Peters A Van Name MA Thorsted BL Piltoft JS Tamborlane WV. Postprandial dosing of bolus insulin in patients with type 1 diabetes: a cross-sectional study using data from the T1d Exchange Registry. Endocr Pract. (2017) 23:1201–9. 10.4158/EP171813.OR 28704103

[41] Evans M Wilkinson M Giannpolou A. Fast-acting insulin aspart: the rationale for a new mealtime insulin. Diabetes Ther. (2019) 10:1793–800. 10.1007/s13300-019-00685-0 31485918 PMC6778592

[42] Ruan Y Elleri D Allen JM Tauschmann M Wilinska ME Dunger DB. Pharmacokinetics of diluted (U20) insulin aspart compared with standard (U100) in children aged 3-6 years with type 1 diabetes during closed-loop insulin delivery: a randomized clinical trial. Diabetologia. (2015) 58:687–90. 10.1007/s00125-014-3483-6 25537835 PMC4351431

[43] Salehi P Roberts AJ Kim GJ. Efficacy and safety of real-life usage of MiniMed 670G Automode in children with type 1 diabetes less than 7 years old. Diabetes Technol Ther. (2019) 21:448–51. 10.1089/dia.2019.0123 31166801

[44] Driscoll KA Volkening LK Haro H Ocean G Wang Y Jackson CC. Are children with type 1 diabetes safe at school? Examining parent perceptions. Pediatr Diabetes. (2015) 16:613–20. 10.1111/pedi.12204 25266418

[45] Lawton J Waugh N Barnard KD Noyes K Harden J Stephen J. Challenges of optimizing glycaemic control in children with Type 1 diabetes: a qualitative study of parents' experiences and views. Diabet Med. (2015) 32:1063–70. 10.1111/dme.12660 25472898

[46] Lawton J Hart R Kimbell B Allen J Beeser R Boughton C. Data sharing whilst using a closed-loop system: qualitative study of adolescents' and parents' experiences and views. Diabetes Technol Ther. (2021). 10.1089/dia.2020.0637. [Epub ahead of print]. PMC8252900 33605790

[47] Lal RA Basina M Maahs DM Hood K Buckingham B Wilson DM. One year clinical experience of the first commercial hybrid closed-loop system. Diabetes Care. (2019) 42:2190–6. 10.2337/dc19-0855 31548247 PMC6868462

[48] Berget CM Laurel H Vigers Tim Frohnert Brigitte I Pyle L. Six months of hybrid closed loop in the real-world: an evaluation of children and young adults using the 670G system. Pediatr Diabetes. (2020) 21:310–8. 10.1111/pedi.12962 31837064 PMC7204168

[49] Rankin D Kimbell B Besser R Boughton C Campbell F Elleri D. Adolescents' experiences of using a smartphone application hosting a closed-loop algorithm to manage type 1 diabetes in everyday life: qualitative study. J Diabetes Sci Technol. (2021). In press. 10.17863/CAM.64591 PMC8411472 34261348

[50] Rankin D Kimbell B Hovorka R Lawton J. Adolescents' and their parents' experiences of using a closed-loop system to manage type 1 diabetes in everyday life: qualitative study. Chronic Illn. (2021):1742395320985924. 10.1177/1742395320985924 33472409 PMC9643806

[51] Forlenza GP Buckingham BA Brown SA Bode BW Levy CJ Criego AB. First outpatient evaluation of a tubeless automated insulin delivery system with customizable glucose targets in children and adults with type 1 diabetes. Diabetes Technol Ther. (2021). [Epub ahead of print]. 33325779 10.1089/dia.2020.0546 PMC8215410

[52] Brown SA Kovatchev BP Raghinaru D Lum JW Buckingham BA Kudva YC. Six-month randomized, multicenter trial of closed-loop control in type 1 diabetes. N Engl J Med. (2019) 381:1707–17. 10.1056/NEJMoa1907863 31618560 PMC7076915

[53] Chernavvsky DR DeBoer MD Keith-Hynes P Mize B McElwee M Demartini S. Use of an artificial pancreas among adolescents for a missed snack bolus and an underestimated meal bolus. Pediatr Diabetes. (2016) 17:28–35. 10.1111/pedi.12230 25348683

[54] Messer LH Berget C Vigers T Pyle L Geno C Wadwa RP. Real world hybrid closed-loop discontinuation: predictors and perceptions of youth discontinuing the 670G system in the first 6 months. Pediatr Diabetes. (2020) 21:319–27. 10.1111/pedi.12971 31885123 PMC7204392

[55] Iturralde E Tanenbaum ML Hanes SJ Suttiratana SC Ambrosino JM Ly TT. Expectations and attitudes of individuals with type 1 diabetes after using a hybrid closed loop system. Diabetes Educ. (2017) 43:223–32. 10.1177/0145721717697244 28340542 PMC7162535

[56] Fuchs J Hovorka R. COVID-19 and diabetes: could diabetes technology research help pave the way for remote healthcare? J Diabetes Sci Technol. (2020) 14:735–6. 10.1177/1932296820929714 32475169 PMC7673188

[57] Quintal A Messier V Rabasa-Lhoret R Racine E. A critical review and analysis of ethical issues associated with the artificial pancreas. Diabetes Metab. (2019) 45:1–10. 10.1016/j.diabet.2018.04.003 29753624 PMC6202257

